# Discovery of *Phytophthora infestans* Genes Expressed in Planta through Mining of cDNA Libraries

**DOI:** 10.1371/journal.pone.0009847

**Published:** 2010-03-24

**Authors:** Roberto Sierra, Luis M. Rodríguez-R, Diego Chaves, Andrés Pinzón, Alejandro Grajales, Alejandro Rojas, Gabriel Mutis, Martha Cárdenas, Daniel Burbano, Pedro Jiménez, Adriana Bernal, Silvia Restrepo

**Affiliations:** 1 Departamento de Ciencias Biológicas, Universidad de los Andes, Bogotá Distrito Capital, Colombia; 2 Dirección de Tecnologías de Información, Universidad de los Andes, Bogotá Distrito Capital, Colombia; 3 Programa de Biología Aplicada, Universidad Militar Nueva Granada, Bogotá Distrito Capital, Colombia; Texas A&M University, United States of America

## Abstract

**Background:**

*Phytophthora infestans* (Mont.) de Bary causes late blight of potato and tomato, and has a broad host range within the Solanaceae family. Most studies of the *Phytophthora – Solanum* pathosystem have focused on gene expression in the host and have not analyzed pathogen gene expression in planta.

**Methodology/Principal Findings:**

We describe in detail an *in silico* approach to mine ESTs from inoculated host plants deposited in a database in order to identify particular pathogen sequences associated with disease. We identified candidate effector genes through mining of 22,795 ESTs corresponding to *P. infestans* cDNA libraries in compatible and incompatible interactions with hosts from the Solanaceae family.

**Conclusions/Significance:**

We annotated genes of *P. infestans* expressed in planta associated with late blight using different approaches and assigned putative functions to 373 out of the 501 sequences found in the *P. infestans* genome draft, including putative secreted proteins, domains associated with pathogenicity and poorly characterized proteins ideal for further experimental studies. Our study provides a methodology for analyzing cDNA libraries and provides an understanding of the plant – oomycete pathosystems that is independent of the host, condition, or type of sample by identifying genes of the pathogen expressed in planta.

## Introduction

*Phytophthora infestans* (Mont.) de Bary causes late blight of potato and tomato, and has a broad host range within the Solanaceae family [1]. This pathogen has been the focus of attention ever since the Irish potato famine because of its devastating effect on economically important crops, causing losses of billions of dollars per year [2], [3]. Although *P. infestans* has been studied for more than a century, little progress has been made on disease control in target host crops [4]. New fungicide-resistant strains are a re-emerging threat to global food security, so the molecular genetics of pathogenicity is now being studied to find alternative approaches that may reduce the use of agrochemicals [5].

Central to plant – oomycete pathosystems is a complex signaling process in which multiple effector proteins are delivered either into the host cell or to the free diffusional space outside the plasma membrane (the host apoplast) to manipulate host cell structure and function [6]. The effector proteins can either promote infection, resulting in benefit to the pathogen, or trigger defensive responses that preclude multiplication of the pathogen [7]. In view of their importance, there is considerable interest in the discovery and characterization of the proteins mediating the host–pathogen interaction. Various classes of effector genes have already been characterized for oomycetes, including the RxLR (for its conserved amino acid motif) family, which currently comprises hundreds of candidate genes [8]–[18]. A second class of effectors, the CRN (for Crinkle and Necrosis) proteins, first identified through an in planta functional expression assay, includes a complex family of relatively large proteins [7], [11], [19]. Finally, there are several apoplastic effectors classified as enzyme inhibitors involved in protection against host defense responses [20]. Schornack et al. (2009) recently reviewed different aspects of the oomycete effectors [21]. The effector secretome of *Phytophthora* is now known to be much more complex than initially expected and is starting to be completely understood thanks to all the progress made during the past few years in this field.

Data mining is one stage in a long–term process of discovery that can be used as a powerful tool to evaluate existing information depending on the researcher's goal. To date, a considerable number of sequences have been obtained from cDNA libraries from *P. infestans* - infected host plants during compatible and incompatible interactions. Some of these sequences encode effector proteins expressed by the pathogen during infection. In previous studies, sequence origin in *P. infestans*–challenged libraries has mainly been analyzed by GC content and/or by sequence similarity [22]. These methodologies lack accuracy because they may overlook sequences belonging to the studied organism or having different GC percentages. The draft of the whole genome of *P. infestans* is now available [11], making it possible to analyze sequence origins precisely within a large data set using bioinformatics tools.

Our goal is to identify *P. infestans* genes expressed in planta through mining of publicly available ESTs corresponding to Solanaceae challenged with *P. infestans* cDNA libraries in compatible and incompatible interactions. To our knowledge, Randall et al. (2005) and Oh et al. (2009) carried out the only studies that have used cDNA interaction libraries to focus on the pathogen's gene expression in planta. Randall et al. (2005) included ca. 5,000 ESTs [3] also included in this study and Oh et al. (2009) screened an interaction library for RXLR discovery and further testing in planta [23]. Our approach allowed us to find interesting genes, including different kinds of effector genes, as candidates for testing in the laboratory. Moreover, we were able to assign putative functions to novel sequences that may provide further understanding of plant–oomycete pathosystems.

## Materials and Methods

### Data Sets

A total of 22,795 ESTs from various libraries constructed from *Solanum* spp. challenged with *P. infestans* were downloaded from GenBank. The accession numbers of the sequences and a description of each library are given in Table 1.

**Table 1:** GenBank accession numbers and descriptions of the sequences used for this study.

Library	GenBank Accn	No. of sequences	Description	Reference
1	EV600946	1	*Solanum tuberosum* ESTs differentially expressed after *Phytophthora infestans*-challenge or DL-beta-amino-butyric acid treatments in detached leaves.	[30]
	EL732250 - EL732349	100		
2	DN154812 - DN154815	4	*P. infestans* induced genes in R-gene-free potato leaves with horizontal resistance 48 hpi.	[31]
	CO267854 - CO267926	73		
3	DR036296 - DR038219	1924	Surface slices of tubers from *S. tuberosum* var. Shepody, infected with *P. infestans* (A2-mating type), 1, 5, 7, 11 and 14 dpi.	[32]
	DN586663 - DN590966	4304		
4	CK640685 - CK640865	181	Differentially expressed genes in a susceptible and moderately resistant potato cultivar Indira and Bettina, respectively.	[33]
	CK656422	1		
5	EG563081 - EG563087	7	cDNA library highly enriched for *P. infestans* repressed genes derived from the moderately resistant potato cv. Bettina 72 hpi.	[34]
6	EG009341 - EG009424	84	cDNA library highly enriched for *P. infestans* induced genes derived from the moderately resistant potato cv. Bettina 24 hpi.	
7	DR751718 - DR752018	301	Suppression subtractive hybridization library of *P. infestans*-challenged *S. tuberosum* detached leaves – Microarrays	[35]
8	BI431351 - BI435900	4548	*P. infestans*-challenged potato leaf, compatible reaction. Whole plants were challenged with 20,000 sporangia/ml of *P. infestans* (isolate US 940480). Leaf tissue was collected at 3, 6, 9, 12, 24, 48, 72 hours after inoculation.	[11]
	BI176280 - BI176417	138		
	BI919288 - BI919361	74		
	BM403790 - BM404085	296		
9	BQ045481 - BQ047783	2303	*P. infestans*-challenged potato leaf, incompatible reaction. Whole plants were challenged with 450,000 sporangia/ml *P. infestans* (isolate US-1 (US940501)). Leaf tissue was collected at 1, 2, 5, 12, and 24 hours post-challenge.	
10	BG589187 - BG592317	3131	*P. infestans*-challenged potato leaf, incompatible reaction. Whole plants were challenged with 450,000 sporangia/ml *P. infestans* (US-1(US 940501)). Leaf tissue was collected at 1, 2, 5, 12, and 24 hours post-challenge.	Zhang, P. et al (2002) unpublished
11	CV969340 - CV969997	658	Infected potato, center of lesion 6 dpi *P. infestans* cDNA.	[3]
12	CV969998 - CV970651	654	Infected potato, outside of lesion 6 dpi *P. infestans* cDNA.	
13	CV965419 - CV969339	3921	Infected tomato, lesion 3 dpi *P. infestans* cDNA.	
14	AJ235735 - AJ235770	36	*S. tuberosum* cv. Stirling genes induced in an early stage of the HR to *P. infestans*.	[36]
15	AJ302109 - AJ302141	33	*S. tuberosum* cv. Bintje leaf genes induced during colonization by *P. infestans*.	[37]
16	AJ437588 - AJ437600	13	Gene expression in two potato lines (*Solanum phureja* x *S. tuberosum* leaf *S. phureja* x *S. tuberosum*) differing in their resistance to *P. infestans* one day after inoculation with *P. infestans*.	[38]
17	AJ487842 - AJ487851	10	*P. infestans* mycelium. Genes regulated during the interaction with potato.	Beyer, K (2002) Unpublished
	**TOTAL**	**22795**		

### Sequence Analysis

**Figure 1:**
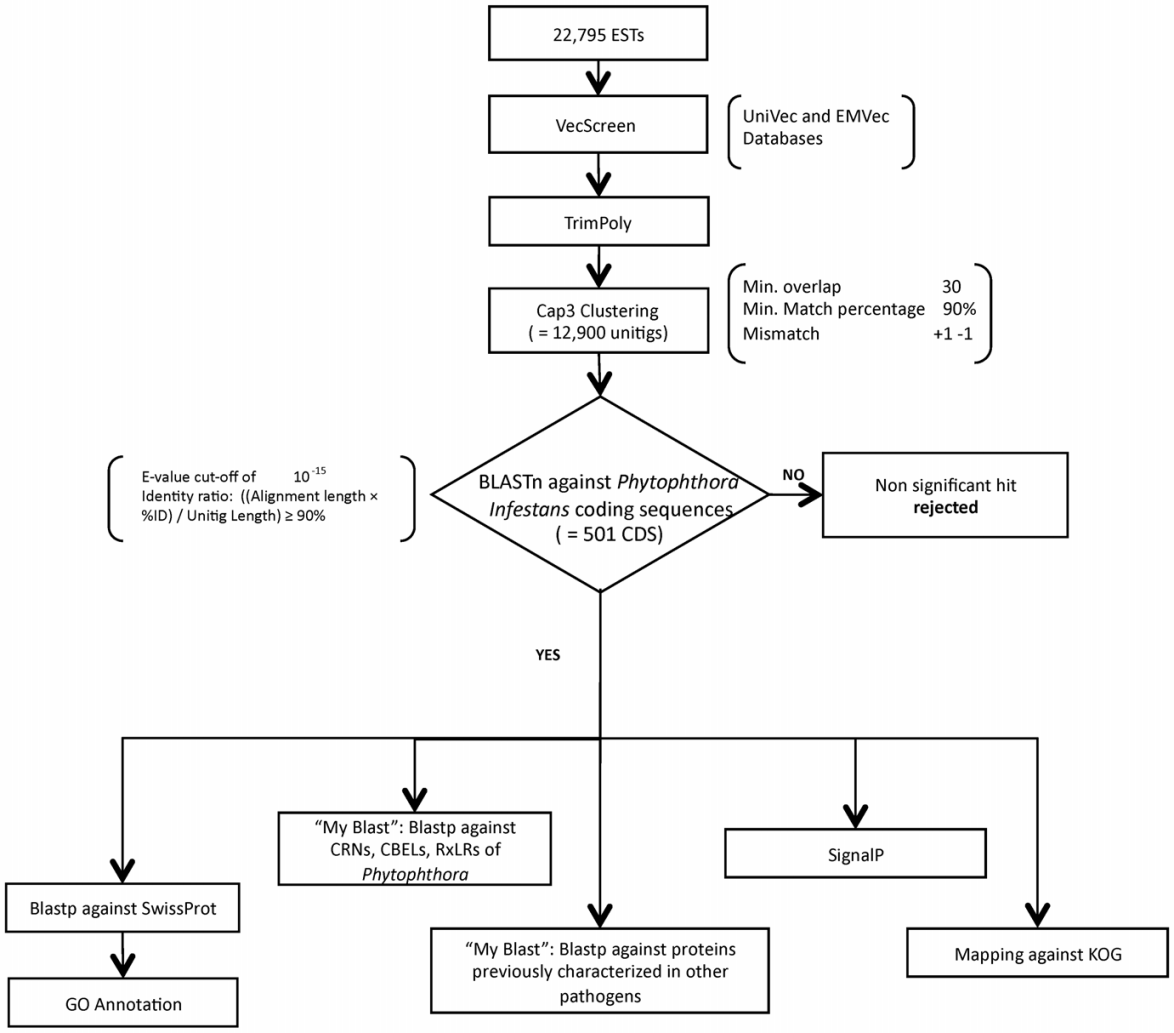
Main workflow of analysis followed in this study.

**Figure 2:**
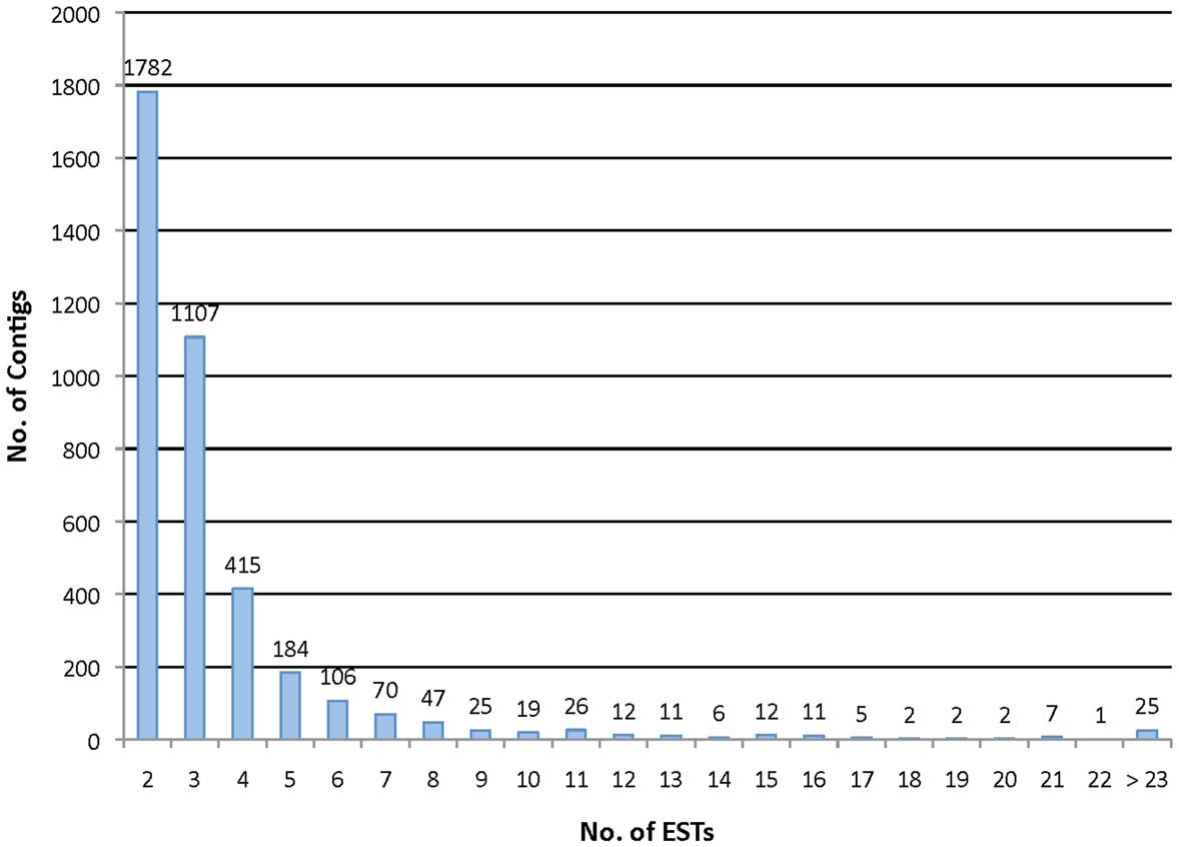
Distribution of ESTs within contigs after clustering (using CAP3) the 22,795 sequences downloaded from GenBank.

The main methodology followed in this study appears in Figure 1. The EST sequences were vector trimmed using VecScreen (http://www.ncbi.nlm.nih.gov/VecScreen/VecScreen.html) with sequential polyA/T and N trimming using the trimpoly program, included in The Institute for Genomic Research (TIGR, currently J. Craig Venter Institute) Gene Indices Seqclean software (http://www.tigr.org/tdb/tgi/software). Clustering was performed using CAP3 [24] (minimum overlap 30; minimum match percentage 90%; mismatch +1 −1) with previous masking using DUST [25] and TGICL [26]. The number of ESTs that were assembled into the resulting contigs was determined (Figure 2).

Similarity searches were conducted for all contigs and singletons (unitigs) using the blastn algorithm [27] with default parameters against the *P. infestans* predicted coding sequences (CDS) accessed through the Broad Institute website retrieved on June 1^st^, 2009 from http://www.broadinstitute.org/annotation/genome/phytophthora_infestans/MultiDownloads.html. The genes were previously predicted from the genome deposited in GenBank accession number AATU01000000 [11]. The unitig hits (*P. infestans* CDS) with an e-value <10^−15^ and an identity ratio of ((Alignment length × %ID)/Unitig length) ≥90% were selected for further analysis.

**Figure 3:**
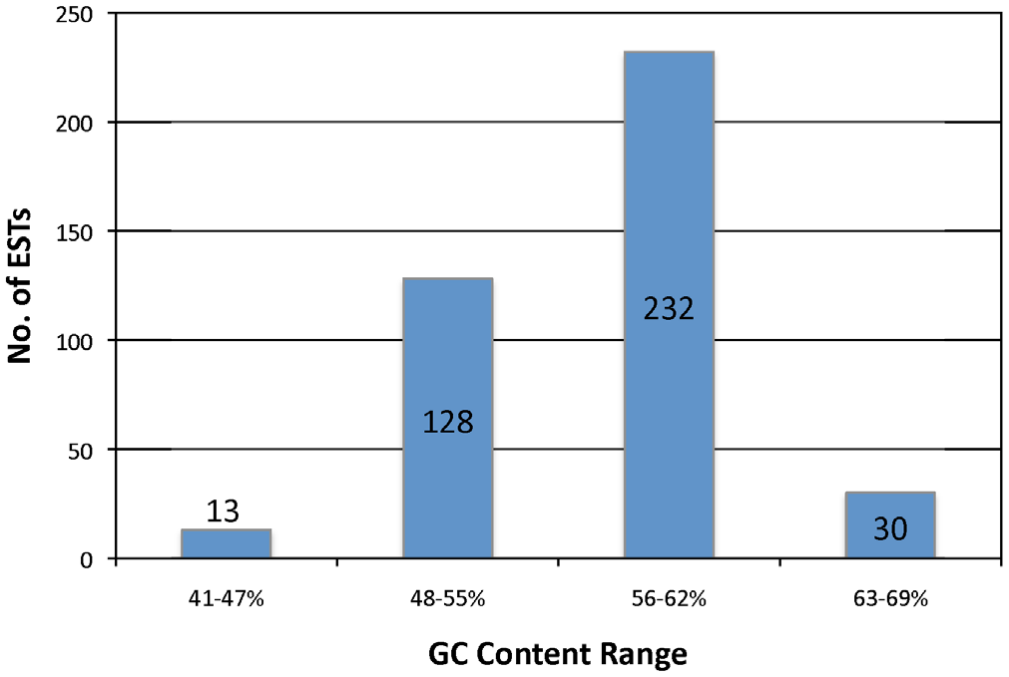
Number of ESTs falling into different GC content ranges among a total of 403 ESTs that had a hit against the *P. infestans* genes.

**Figure 4:**
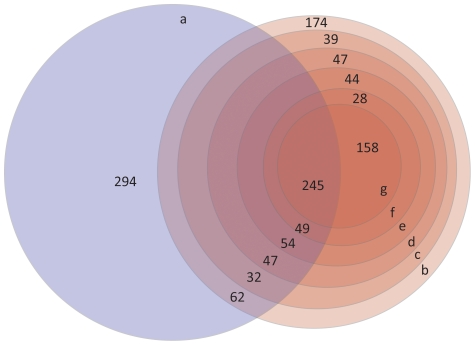
Different criteria used to separate the *P. infestans* sequences from host sequences produces results that differ notably among them. 12,900 unitigs containing host and pathogen sequences were used to test different approaches to separate both types of sequences. The scheme represents the number of sequences obtained using GC content and a BLAST cut-off e-value combined with different ratio cut-offs: ((Alignment length * %ID)/Unitig length). (a) GC >52%; (b) e-value cut-off of 10^−15^ (c) e-value cut-off of 10^-15^and ratio >50%; (d) e-value cut-off of 10^−15^ and ratio >60%; (e) e-value cut-off of 10^−15^ and ratio >70%; (f) e-value cut-off of 10^−15^ and ratio >80%; (g) e-value cut-off of 10^−15^ and ratio >90%.

The GC contents of the 403 unitigs that had a hit against the *P. infestans* CDS were calculated using the EMBOSS tool Geecee [28] (Figure 3), as well as for all the 12,900 unitigs. Additionally, the unitigs were filtered using a GC content cut-off of >52% as previously reported [3], [22], by e-value (cut-off of <10^−15^) against the *P. infestans* CDS [11], and by applying the same e-value cut-off and different identity ratio (as above) percentages (>50, >60, >70, >80, or >90%) (Figure 4). The resulting sequences were analyzed with the blastn algorithm against the potato (*Solanum tuberosum*) and tomato (*Solanum lycopersicum*) EST sequences and against the Solanaceae ESTs deposited in the GenBank dbEST to test whether the identity ratio cut-off was being useful to separate host from pathogen sequences (Table 2).

**Table 2:** Summary of resulting candidate *Phytophthora infestans* sequences after separating 12,900 unitigs containing pathogen and host sequences using different selection criteria.

Cut-off Criteria					Sequences with hits (e-value <10^−30^) (No.)		Sequences with hits (e-value <10^−30^) (%)	
Criterion	E-value (against *P. infestans* CDS)	Ratio (against *P. infestans* CDS)	%GC	No. of significant hits	Against ESTs of Potato & Tomato	Against ESTs of Solanaceae	Against ESTs of Potato & Tomato	Against ESTs of Solanaceae
GC	Any	Any	>52%	783	153	234	19.54	29.89
E-value	<10^−15^	0	Any	979	199	285	20.33	29.11
E-value + Ratio50	<10^−15^	>50%	Any	743	107	177	14.40	23.82
E-value + Ratio60	<10^−15^	>60%	Any	672	99	164	14.73	24.40
E-value + Ratio70	<10^−15^	>70%	Any	578	83	138	14.36	23.88
E-value + Ratio80	<10^−15^	>80%	Any	480	65	114	13.54	23.75
E-value + Ratio90	<10^−15^	>90%	Any	403	51	86	12.66	21.34

The sequences identified as belonging to *P. infestans* but also having a hit against potato and tomato ESTs or any Solanaceae are shown to the right.

a ((Alignment length * %ID)/Unitig length).

**Figure 5:**
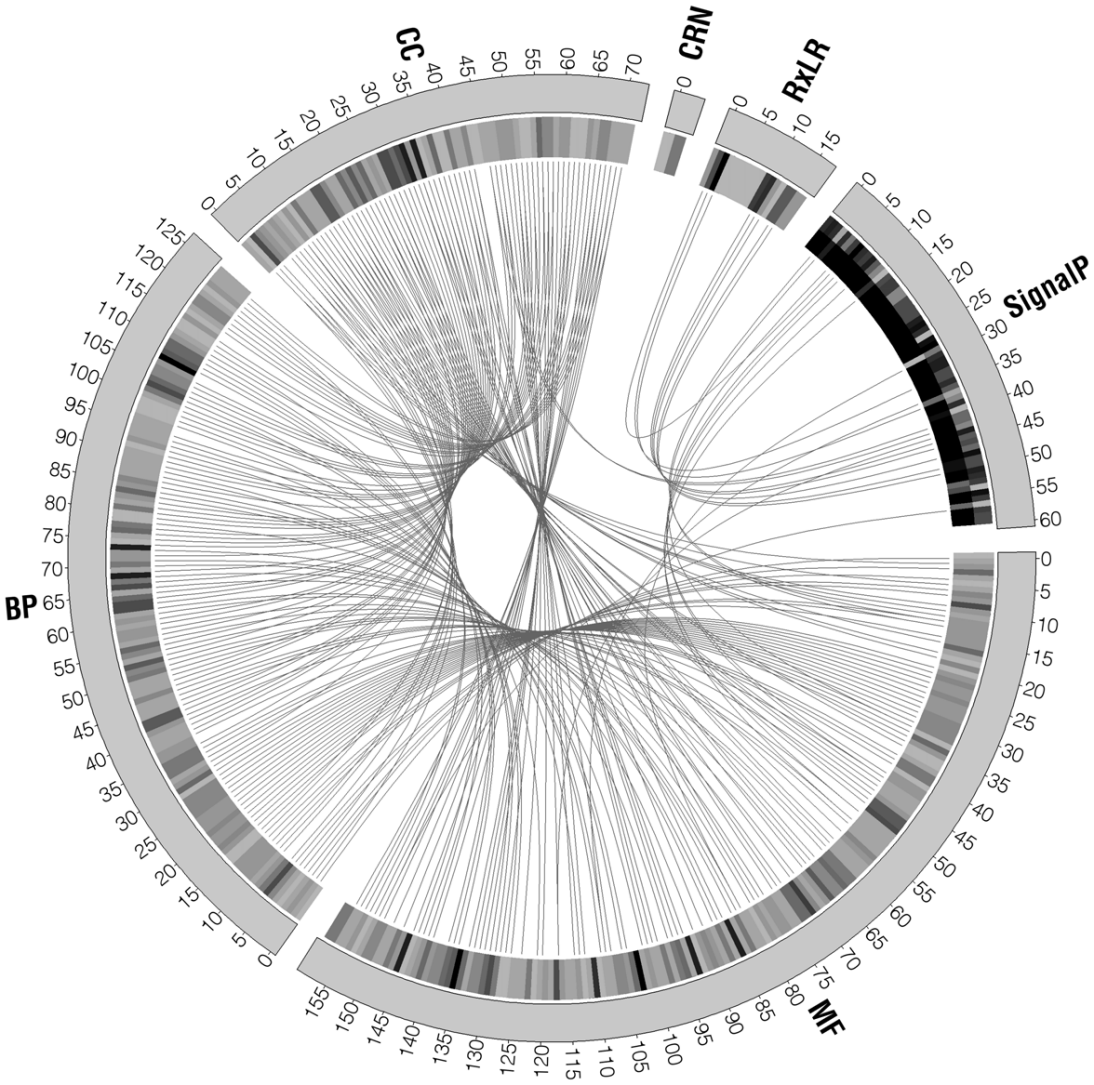
Visualization of the sequence annotation using GO categories, BLAST results and SignalP using the Circos software. Blank spaces (i.e. not linked) show sequences annotated by one approach only, one link (connection line) shows it was annotated by two different approaches and so on. The inner circle in a scale of grays shows the bit scores for the BLAST results and the D value and probability S for SignalP results, darker marks show better scores. MF: Molecular Function according to GO categories, BP: Biological Process according to GO categories, CC: Cellular Component according to GO categories, RXLR: BLAST hits against the RXLR database, CRN: Blast hits against the CRN database, and SignalP: secretion peptide results using SignalP.

**Figure 6:**
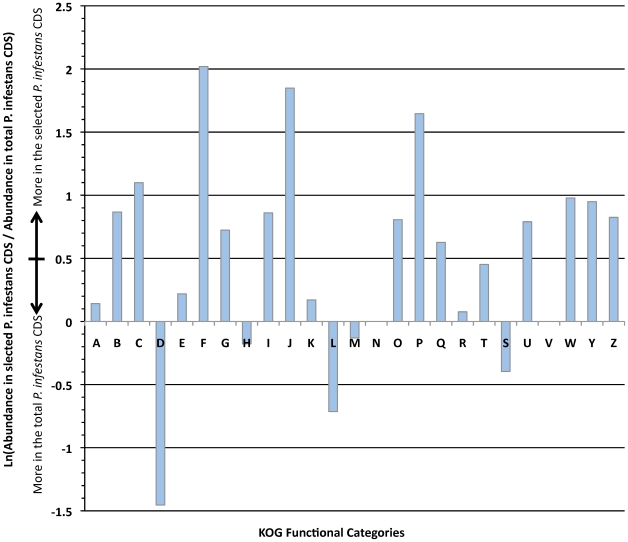
Differential abundance of predicted CDS found in the 501 selected sequences and the total predicted *P. infestans* CDS, based on KOG functional categories. The differential abundance (*y* axis) of predicted CDS to assignable categories (*x* axis) is shown. KOG categories are as follows (from: http://www.ncbi.nlm.nih.gov/COG/): J, Translation; A, RNA processing and modification; K, Transcription; L, Replication, recombination and repair; B, Chromatin structure and dynamics; D, Cell cycle control, cell division, chromosome partitioning; Y, Nuclear structure; V, Defense mechanisms; T, Signal transduction mechanisms; M, Cell wall/membrane/envelope biogenesis; N, Cell motility; Z, Cytoskeleton; W, Extracellular structures; U, Intracellular trafficking, secretion, and vesicular transport; O, Posttranslational modification, protein turnover, chaperones; C, Energy production and conversion; G, Carbohydrate transport and metabolism; E, Amino acid transport and metabolism; F Nucleotide transport and metabolism; H, Coenzyme transport and metabolism; I, Lipid transport and metabolism; P, Inorganic ion transport and metabolism; Q, Secondary metabolites biosynthesis, transport and catabolism; R, General function prediction only; S Function unknown.

The 501 *P. infestans* CDS selected based on the blastn results of unitigs with significant hits (e-value <10^−15^ and identity ratio >90%) were searched against the SwissProt [29] database (word size  = 3, matrix  =  BLOSUM62) using blastp [30] to provide a putative function. All of the sequences that had a hit against the SwissProt database were assigned to a protein family based on the Pfam database (data not shown) and based on these were assigned to a Gene Ontology (GO) category (GO database release July, 2009) [31] (Figure 5). The selected 501 predicted *P. infestans* proteins as well as the complete set of previously predicted proteins from the *P. infestans* genome by Haas et al. (2009) were mapped against the KOG database by in house scripting. Both results were compared in terms of abundance as shown in Figure 6. The best BLAST matches were selected based on e-value and sequence coverage at each KOG category.

Small databases containing *Phytophthora* CRNs, RXLRs, CBELs (for cellulose-binding (CB), elicitor (E) of defense in plants and lectin-like (L) activities) and suppressor of necrosis genes were created from the non-redundant protein database in GenBank, as well as databases with genes previously characterized to be involved in pre-penetration, penetration, appresorium and haustorium formation from *Magnaporthe oryzae*, (formerly *Magnaporthe grisea*), *Colletotrichum* spp. and *Uromyces* spp. All of the custom made databases are publicly available at http://bioinf.uniandes.edu.co/pi/MiniDB.zip. Similarity searches were conducted for the selected 501 *P. infestans* CDS that had a hit against the initial unitigs using the blastp algorithm [27] with default parameters against each data sets using the “myBlast” tool (http://bioinf.uniandes.edu.co/rblast.php). All the sequences were annotated according to their best BLAST hit against any of the databases mentioned above. All of these sequences were predicted for the presence and location of signal peptide cleavage sites with SignalP 3.0 [32]. The results for blastp (against the RXLRs and CRNs database), SignalP and GO annotation (molecular function, biological process and cellular component) were graphed with the Circos software [33] to show the amount of sequences that could be annotated using one or more approaches. Integrating the information in this software allows the researcher to visually find genes that meet certain criteria of interest in an easy way.

## Results

A total of 12,900 contigs and singletons (unitigs) were obtained after clustering a set of 22,975 ESTs into contigs from the 17 libraries (Table 1). The set of unitigs is comprised of 3,877 contigs and 9,023 singletons. The percentage of redundancy of the ESTs assembled into contigs was 60.4%. The contigs were mostly composed of two to five ESTs (Figure 2), with a maximum of 184 different ESTs composing a single contig (data not shown).

The sequences (ESTs) composing the unitigs of interest (i.e. having a hit against the *P. infestans* CDS) were mapped back to the libraries of origin to describe from which libraries were these sequences coming from. This would allow determining if there were a tendency for ESTs to cluster together among ESTs from the same library or randomly among libraries. From the 403 unitigs that had a hit against the *P. infestans* CDS, 61 were contigs composed of sequences from libraries 7, 8, 9, 10, 11, 12, and 13 (Table 1). The sequences from the tomato library (library 13) clustered together in 23 contigs and with sequences from potato libraries (libraries 7, 10, and 11). A total of 240 unitigs were unique to the tomato cDNA library. The GC content of the 403 unitigs of interest was calculated as described above. The average GC content was 57% ranging from 41–69% (Figure 3), the entire *P. infestans* genome has about 51% GC content [11], and previous studies reported an average of 57% for the GC content of *P. infestans* ORFs [19].

When filtering the 12,900 unitigs by GC content >52%, 783 unitigs resulted as *P. infestans* candidate sequences. These sequences were searched against the potato and tomato ESTs resulting in 153 queries sequences (19.5% of the selected sequences) with a significant hit (Figure 4a and Table 2). If an e-value criteria of <10^−15^ against the *P. infestans* CDS [11] is used, more sequences are recovered (979 unitigs) but 20.3 and 29.1% of these sequences also have a hit against potato and tomato or Solanaceae ESTs, respectively (Figure 4b and Table 2). After applying the e-value (<10^−15^) criteria against the *P. infestans* CDS combined with different identity ratio percentages, the number of unitigs obtained was reduced with more stringent criteria, as expected (Figure 4c, d, e, f, g and Table 2). Nonetheless, if using an e-value and identity ratio cut-off of >90% the number of hits against Solanaceae ESTs declined 8.6 percentual points compared with a GC content criterion (Figure 4g and Table 2).

To annotate the *P. infestans* genes expressed during infection, we used the hits (*P. infestans* CDS [11]) resulting from the blastn analysis. A total of 403 (3.12%) unitigs from *P. infestans*-challenged plants had a hit against 532 *P. infestans* genes, from 22,658 predicted genes by Haas et al. (2009), and these unitigs were distributed in 61 contigs and 342 singletons. From the 532 *P. infestans* CDS, 31 sequences had redundant hits and as a result 501 sequences were selected as unique hits (or subject sequences) and used for further annotation.

169 out of the 501 *P. infestans* CDS are classified as hypothetical proteins in the Broad Institute and do not have significant hits against SwissProt. 41 of these sequences were found to have a secretion signal (based on SignalP), a hit against a RXLR, CRN (Figure 5), CBEL, or *Magnaporthe oryzae* gene (data not shown). From the other 332 sequences, the Broad Institute classifies 245 sequences as hypothetical proteins despite there is at least one significant hit against SwissProt, and assigns a function to 87 sequences, from which we found the corresponding annotations within the SwissProt hits (e.g. 30 sequences were in agreement as ribosomal proteins). Of the 332 sequences with hits against SwissProt, 293 were linked to a protein family according to Pfam (data not shown) and these results were used to map them to gene ontology (GO) categories resulting in 159 assigned to molecular function, 129 to biological process and 72 to cellular process. Any given CDS defined in the GO database could be assigned to more than one ontology (Figure 5).

Within these 501 sequences we found sequences with high similarity to 16 *Phytophthora* CRNs, 17 RXLRs, and 1 CBEL. Although the best BLAST hits resulted in 3 CRNs, the 16 characterized CRNs [7], [19] were found within the high-scoring segment pairs (HSPs) of these BLAST hits. Additionally, these sequences were not found in the SwissProt database or had any other annotation (Figure 5). Since It has been shown that the bit scores in BLAST result are more explanatory than e-values [34], due to the fact that the e-value depends on the size of the database used, we chose a bit score of 100 as the cut-off value for blast results when any of the small databases (described above) were used.

14 and 31 of these 501 sequences had a hit against the genes previously characterized as involved in pathogenesis for *Colletotrichum* spp. and *M. oryzae*, respectively (data not shown). We identified *P. infestans* genes with high similarity to *Colletrotrichum* spp. genes involved in appresorium formation (5 genes) and conidia germination and appresorium formation (2 genes). Similarly, with *M. oryzae* genes involved in apresorium formation (13 genes), acting as pathogenicity factor (9 genes) and infection-related mophogenesis (3 genes), among others.

Furthermore, 60 sequences had a secretion signal based on a combination of several artificial neural networks and hidden Markov models incorporated in SignalP [32], both models had to be in agreement for a secretion signal in order for us to consider it a positive sequence for secretion. Interestingly, some of the leader sequences of *P. infestans* effector proteins containing the RXLR motif identified in oomycetes [10], [12], [15], [35] can be seen as also having a secretion signal according to SignalP (Figure 5). These sequences are categorized as hypothetical proteins in the Broad Institute database and are intriguing sequences for further study in a plant – *Phytophthora* recognition model since these may be novel genes in the interaction.

The 501 selected *P. infestans* predicted CDS and the complete set of 22,658 P. infestans predicted CDS were mapped to the KOG database assingning 299 and 8,622 to at least one category, respectively. The abundance of these sequences in the KOG categories was compared as shown in Figure 6. Multifasta files with the resulting sequences from the different analysis as well as sequences that could not be annotated can be downloaded from http://bioinf.uniandes.edu.co/pi/Resulting_sets.zip.

The Circos software [33] was used to visualize all data resulting from blastp, SignalP and gene ontology annotations showing how every *P. infestans* sequence could be annotated with one, two or up to six different approaches. Results of how the sequences interlace with the different annotation approaches can be seen in Figure 5.

## Discussion

This study attempts to extract genes from cDNA libraries expressed from the *P. infestans* transcriptome during attack on the host, using a combination of available resources and an innovative bioinformatics approach. First, our raw data consisted of all the sequences available to date from transcriptomic studies of Solanaceae – *Phytophthora* interactions. Secondly, we took advantage of the most recent release of the *P. infestans* genome to separate pathogen from host sequences. Thirdly, the annotation process was exhaustive, using similarity approaches with a curated database and other small databases that contained characterized genes involved in pathogenesis, improving the efficiency of the whole annotation.

We were able to test our methodology with different cut-off criteria based on GC content, e-value and an identity ratio (Table 2). The different methodologies showed that an e-value criterion by itself would be too permissive allowing almost 30% of the selected sequences to match different organisms. The GC content cut-off showed to be more stringent than e-value criterion but similarly permissive as the e-value criterion. On the other hand, a combined strategy of e-value and identity ratio percentages seemed to be more useful. In this study we used strict cut-off values to reduce the amount of host sequences and thus provide a relatively small set of candidate sequences for further testing in the laboratory.

A total of 3.12% of the initial unitigs assembly from interaction library of ESTs had a hit against the *P. infestans* genome. We knew that *P. infestans* sequences were present in the challenged libraries [22] but we did not know whether there was a representative number to analyze the gene expression of *P. infestans*. In previous studies, the estimation of “contaminating” pathogen sequences was based on GC content [22], [36]. In view of the broad range of GC contents in the *P. infestans* CDS analyzed (ESTs), it is clearly difficult to separate sequences belonging to host and pathogen merely by GC content. Since the GC content of potato is in average 42,7% [22], previous studies could have underestimated the “contamination” with *P. infestans* sequences. After cleaning their sequences by GC content, Ronning et al. (2003) stated that there may still be “contaminating” *P. infestans* sequences from the compatible (library 8) and incompatible (library 9) libraries. From these same libraries (8 and 9) we obtained seven and five ESTs, respectively, that had hits against the *P. infestans* predicted genes, with highly stringent parameters (see materials and methods).

Mapping against the KOG categories showed that more expressed categories found on selected proteins were nucleotide transport and metabolism (F), translation, ribosomal structure and biogenesis (J) and inorganic ion transport and metabolism. Cell cycle control, cell division and chromosome partitoning (D), is clearly biased on the pathogen genome as expected, since this type of regulations are fundamental for pathogen growth and development and maintenance and its role in pathogenicity is hard to interpret. This situation can also be evidenced by the bias in replication, recombinantion and repair categorie (L) at the genome level.

The previously uncharacterized genes may also be involved in signalling pathways of vital importance for understanding the *Solanum-Phytophthora* interaction and would require further study to conclude their role during infection. According to microarray gene expression data there are at least 494 genes differentially expressed during infection and some have been identified as involved in haustorial formation in early stages and in mycelial necrotophic growth in the latter stages [11]. We have identified genes with high similarity to genes involved in conidia germination and appresorium formation in the *Colletotrichum* spp. model. Knowing the putative function of these genes makes them interesting as candidates to characterize in the laboratory to identify their function in the *Solanum-Phytophthora* pathosystem and.

In the current genomics era of low-cost DNA sequencing and high–throughput techonologies, enormous amounts of data at the DNA and RNA level are being produced daily. However, if this is not coupled with high-throughput methods of annotation, we are diminishing its real potential. It is important to note that, although this workflow analysis was applied to the *Phytophthora – Solanum* interaction, it may also be applied to any other host–microbe interaction for which sufficient data have been generated, as is the case for pathosystems of clinical and agricultural importance. Finally, we intend that our innovative methodology will be used in other studies in such a way as to recover useful data from databases and contribute to new findings in different areas of expertise.
